# Design of a randomised, placebo-controlled, double-blind multicentre study assessing the effect of colchicine on the incidence of knee or hip replacements in symptomatic knee or hip osteoarthritis: the ECHO trial

**DOI:** 10.1136/bmjopen-2024-098096

**Published:** 2025-04-14

**Authors:** Michelle W J Heijman, Cornelia H M van den Ende, Jan H Cornel, José M H Smolders, Henk J Schers, Wietske Kievit, Sander Koeter, Bart J F van den Bemt, Calin D Popa

**Affiliations:** 1 Department of Research, Sint Maartenskliniek, Nijmegen, Netherlands; 2 Department of Rheumatology, Radboud University Medical Center, Nijmegen, Netherlands; 3 Department of Cardiology, Radboud University Medical Center, Nijmegen, Netherlands; 4 Department of Cardiology, Northwest Clinics, Alkmaar, Netherlands; 5 Dutch Network for Cardiovascular Research (WCN), Utrecht, Netherlands; 6 Department of Orthopedics, Sint Maartenskliniek, Nijmegen, Netherlands; 7 Department of Primary and Community Care, Radboud University Medical Center, Nijmegen, Netherlands; 8 Department of Health Evidence, Radboud University Medical Center, Nijmegen, Netherlands; 9 Department of Orthopedics, Canisius Wilhelmina Hospital, Nijmegen, Netherlands; 10 Department of Pharmacy, Sint Maartenskliniek, Nijmegen, Netherlands; 11 Department of Pharmacy, Radboud University Medical Center, Nijmegen, Netherlands; 12 Department of Rheumatology, Sint Maartenskliniek, Nijmegen, Netherlands

**Keywords:** RHEUMATOLOGY, Hip, Knee, Drug Therapy, Randomized Controlled Trial, Inflammation

## Abstract

**Introduction:**

Osteoarthritis (OA) is a multifactorial disease in which low-grade inflammation is considered to play a pivotal role. Although colchicine is a widely used anti-inflammatory drug in the treatment of gout, its effect in OA is still disputed due to inconsistent results of short-term clinical trials. Therefore, we aim to evaluate the effect of long-term colchicine 0.5 mg once daily on the incidence of knee or hip replacements in patients with knee or hip OA.

**Methods and analysis:**

The ECHO trial is a prospective, multicentre, randomised, double-blind, placebo-controlled, phase III trial in which 1200 participants with knee or hip OA tolerant to colchicine during a 30-day run-in period will be 1:1 randomised to colchicine 0.5 mg once daily or matching placebo using concealed allocation. The primary endpoint is the time from randomisation to the first knee or hip replacement assessed up to 4.5 years. Secondary endpoints include course of pain, physical function, joint space narrowing, low-grade inflammation, quality of life, clinical or radiological onset of OA in a new joint group other than present at baseline, number of participants using pain medication during the study, onset of new cardiovascular events (ie, myocardial infarction, ischaemia-driven coronary revascularisation, ischaemic stroke, peripheral artery disease or cardiovascular death) and direct and indirect costs related to treatment and disease burden due to OA. Harm-related endpoints include the number of (serious) adverse events, the number of withdrawals due to (serious) adverse events and changes in laboratory data (ie, serum creatinine, estimated glomerular filtration rate and alanine transferase) throughout the study. The primary analysis will be performed according to the intention-to-treat principle.

**Ethics and dissemination:**

This trial has been approved by the Medical Ethics Review Committee East-Netherlands. Findings will be presented at scientific meetings and published in a peer-reviewed scientific journal.

**Trial registration number:**

NCT06578182.

**STRENGTHS AND LIMITATIONS OF THIS STUDY**

- This is the first randomised controlled trial with low-dose, long-term treatment of colchicine in a large number of patients with osteoarthritis recruited from multiple centres in the Netherlands.
- The primary endpoint is the time from randomisation until the date of first documented knee or hip replacement, date of death, date of loss to follow-up or study end-date, whichever comes first, assessed up to 4.5 years.
- The adaptive design of this event-driven trial allows for a blinded re-estimation of the sample size.
- Adherence to trial medication may subside over time.

## Introduction

 Osteoarthritis (OA) is one of the leading causes of pain and disability, currently affecting more than 500 million people worldwide.^1^ Towards 2050, it is estimated that the number of OA patients will globally rise by 60–100% along with increases in the prevalence of obesity and longevity.^2^ Without any disease-modifying osteoarthritic drugs (DMOADs) available, a substantial challenge to healthcare systems is posed.^3^

The pathophysiology of OA is multifactorial and a low-grade chronic inflammation, indicative of an inflammatory phenotype, has been described in the affected joints.^4 5^ This inflammation is mediated primarily by the innate immune system. A critical component of the innate immune system is the NOD-like receptor protein 3 (NLRP3) inflammasome, which mediates caspase-1 activation and the secretion of proinflammatory cytokine interleukin 1 beta (IL-1β).^6^ IL-1β is considered one of the major players implicated in the pathogenesis of OA, by both activating innervating nociceptors and promoting joint destruction via catabolic proteins such as matrix metalloproteinases (MMPs).^7^ This suggests that therapeutically targeting this pathway in OA may potentially prevent or reduce cartilage destruction and pain, thereby slowing the progression of the disease.

An exploratory analysis in the canakinumab anti-Inflammatory thrombosis outcomes study involving patients with a history of myocardial infarction has recently supported this hypothesis.^8^ Inhibiting IL-1β with canakinumab reduced the rates of total knee replacements and total hip replacements during a median follow-up of 3.7 years (HR, 0.58; CI 0.42 to 0.80).^9^ Due to the high cost of canakinumab; however, further exploration of affordable anti-inflammatory therapies with similar properties for the treatment of OA is warranted.

Colchicine, an alkaloid extracted from the autumn crocus (*Colchicum autumnale*), has been widely used in acute crystal-induced inflammation and gout flare prophylaxis.^10^ By binding to tubulins, it prevents microtubules from assemblage and polymerisation. This results in disrupted microtubule function and broad cellular actions including inhibition of the NLRP3 inflammasome and MMP13.^11^ Hence, there is also reason to assume that colchicine may slow the progression of OA.

Up to now, three randomised controlled trials with follow-ups of less than 6 months using colchicine 0.5 mg twice daily alone or in combination with nimesulide or piroxicam versus placebo alone or in combination with nimesulide or piroxicam demonstrated symptomatic benefits regarding pain, function and global assessment in patients with OA.^12^,^15^ These results were confirmed by another randomised controlled trial using colchicine 1.5 mg once daily in combination with paracetamol versus paracetamol alone.^16^ Moreover, in a non-randomised controlled trial, administration of colchicine 0.5 mg twice daily in combination with paracetamol resulted in stable levels of cartilage oligomeric matrix protein (COMP), suggesting the stability of the cartilage.^17^ When paracetamol was used alone, the levels of serum COMP significantly increased from 2 months to 1 year. The results of this study indicate that colchicine might act as a DMOAD by stabilising cartilage turnover and preventing further degradation.

In contrast, in the colchicine effectiveness in symptoms and inflammation modification in knee OA (COLKOA) study, no statistically significant difference in knee OA symptoms was seen between colchicine 0.5 mg twice daily and placebo over 16 weeks.^18^ Nevertheless, COLKOA did find that colchicine reduced systemic inflammation based on high-sensitivity C reactive protein (hs-CRP) and bone turnover based on cross-linked C-telopeptide of type I collagen, which are both biomarkers that are associated with the progression of OA. Furthermore, colchicine taken 0.5 mg twice daily over 3 months failed to improve symptoms in patients with OA in the hands compared with placebo in two randomised controlled trials, but one of these trials may have been underpowered and was likely diluted by non-inflamed OA subjects.^19^,^21^ Finally, comparing the efficacy of 1 mg/day colchicine treatment versus 16 weeks of physical therapy in 62 patients with knee OA showed that physical therapy was more effective than colchicine in reducing pain and improving physical function.^22^

Due to these contradictory results, there is currently not enough evidence to recommend colchicine as a treatment for knee or hand OA.^12 23^ However, it is important to note that each study included no more than 150 patients with follow-up periods not exceeding 1 year. To study the long-term effects of colchicine, a post-hoc analysis of the low-dose colchicine 2 (LoDoCo2) trial was recently performed in which 5522 patients with evidence of coronary disease were randomly assigned to receive colchicine 0.5 mg once daily or matching placebo over a median follow-up of 28.6 months.^24^ Based on the adverse event (AE) data, colchicine 0.5 mg daily was associated with a lower incidence of total knee or hip replacements as compared with placebo (HR 0.69; CI 0.51 to 0.95).^25^ Long-term safety data from this randomised controlled trial did not show an increase in life-threatening or serious AEs (SAEs).^26^ Further investigation of long-term therapy with colchicine to slow disease progression in OA is needed, as the LoDoCo2 trial was not designed for this purpose.

Therefore, we aim to evaluate the effect of long-term use of colchicine 0.5 mg once daily compared with placebo in patients with knee or hip OA on the incidence of knee or hip replacement throughout 3–4.5 years.

## Methods and analysis

This protocol has been reported following the Standard Protocol Items: Recommendations for Interventional Trials guidelines (online supplemental Additional file 1).^27^

### Study design

**Figure 1:**
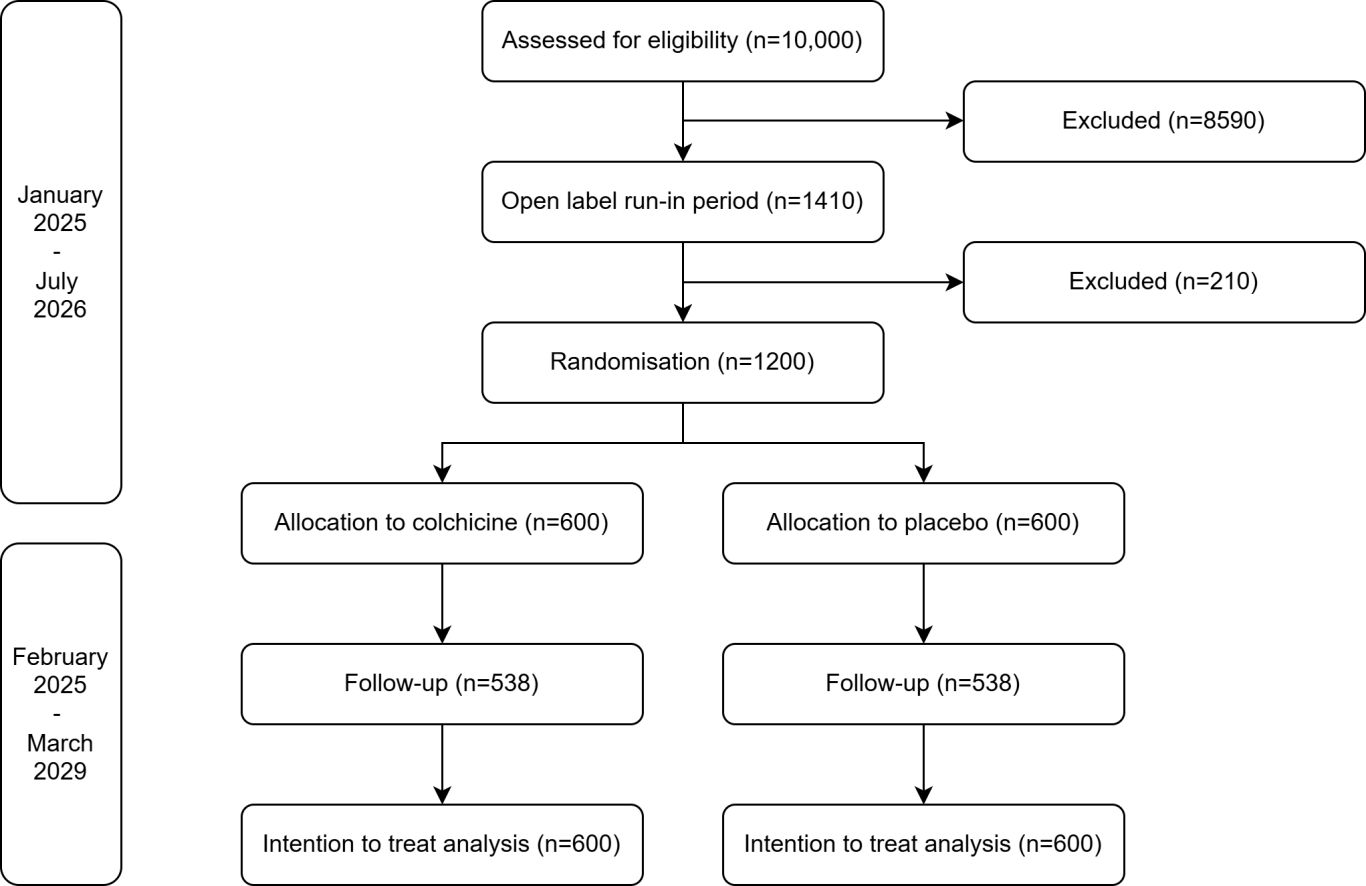
Flowchart of the study.

The ECHO trial is designed as a multicentre, randomised, placebo-controlled, double-blind, event-driven superiority trial. After signing informed consent, all eligible patients will use colchicine 0.5 mg once daily for 30 days. Patients without AEs, maintaining adherence and expressing continued willingness to participate after this open-label run-in period will be randomly allocated in a 1:1 ratio to colchicine or placebo. Depending on the time of inclusion (planned between January 2025 and July 2026), the trial duration for each patient will range from 3 years to 4.5 years, as the study end date is approximately the same for all participants (estimated at March 2029). A flowchart is shown in figure 1.

### Study population

To be eligible to participate in this study, participants must meet all of the following criteria: (1) clinical diagnosis of knee or hip OA; (2) aged between 45 years and 80 years; (3) at least 2-year history of complaints due to OA in the hip and/or knee or documented radiographic changes typical for advanced knee and/or hip OA (Kellgren and Lawrence (KL) score ≥2). A potential participant who meets any of the following criteria will be excluded from participation: on a waiting list for primary joint replacement surgery of the hip or knee, irrespective of cause, any absolute contraindication for knee or hip replacement in the future, more than one previous hip or knee replacement, other known medical disease that may affect joints, known generalised pain syndromes such as fibromyalgia, renal impairment as evidenced by serum creatinine >150 µmol/L or estimated glomerular filtration rate (eGFR) <50 mL/min/1.73 m^2^, liver function impairment as evidenced by serum alanine transferase (ALAT) >3 upper limit of normal (ULN), blood dyscrasia, high frailty (clinical frailty scale ≥7) or predicted life expectancy <5 years, peripheral neuritis, myositis or marked myo-sensitivity to statins, current use of colchicine for another indication, intolerance to colchicine, use of macrolide antibiotics (ie, clarithromycin, erythromycin and azithromycin), antimycotics (ie, ketoconazole, itraconazole and voriconazole), protease inhibitors and antiretroviral drugs (ie, ritonavir, lopinavir, tipranavir, atazanavir, darunavir, indinavir, saquinavir and cobicistat), antiarrhythmic drugs (ie, verapamil and diltiazem) or immunosuppressants (ie, cyclosporine), current enrolment in another trial, incapacitated patients, pregnant or breastfeeding women, fertile female participants not taking sufficient contraception or male participants unwilling to use effective contraception during the study to prevent pregnancy.

### Recruitment and screening procedures

Potentially eligible patients visiting the department of orthopaedics or rheumatology from the referral and participating centres in the Netherlands will be informed by their physicians (online supplemental additional file 2). In addition, the general practitioner practices of the primary care practice-based research network of Radboudumc Nijmegen, ReumaNederland (a national patient organisation and rheumatism research funder) and Poly-Artrose Lotgenoten (P-AL; a national patient organisation) will disseminate information on the study and invite people with knee or hip OA to show their interest.

Patients who express their interest will be invited to complete an online questionnaire on a secure website (Castor electronic data capture (EDC)) to assess elementary eligibility criteria and provide their consent to be contacted by a member of the research team. Individuals who had previously expressed their interest before the study’s commencement due to national publicity related to our previously published findings of the LoDoCo2 trial will similarly be approached.^25^ Those deemed potentially eligible will then undergo further screening by phone and will be asked for their consent to contact their treating physician to confirm the diagnosis, send recent X-rays of hip or knee joints if present and gather additional eligibility information if needed.

Potentially eligible patients who express their willingness to participate during the phone call will undergo a screening visit at one of the locations of the participating centres in the Netherlands. Following informed consent, blood samples will be collected to assess liver and renal function, and all participants will be supplied with open-label colchicine 0.5 mg once daily for 30 days (run-in period). Participants will be instructed to commence medication after reviewing laboratory results. If intolerance to colchicine is suspected (eg, gastrointestinal upset), patients are instructed to stop therapy immediately and encouraged to rechallenge themselves after 5 days. If symptoms initially resolve but reoccur when the drug is reintroduced, they will be assumed to be intolerant to colchicine. In addition, if the eGFR >50 mL/min/1.73 m^2^ or serum ALAT <3 ULN at the start of the run-in phase, but <50 mL/min/1.73 m^2^ or > 3 ULN at baseline, patients will not be randomised.

Patients without AEs, who maintain adherence and express a continued willingness to participate after the run-in period will proceed to the baseline visit. Blood samples will be obtained to assess liver and renal function, and an X-ray of the index joint will be taken to assess KL score and joint space narrowing if not taken in the past 6 months.

### Randomisation, blinding and treatment allocation

Eligible patients will be randomised to colchicine 0.5 mg once daily or placebo with an allocation ratio of 1:1. Randomisation will be performed by an independent pharmacist of the Sint Maartenskliniek using block randomisation with variable block sizes of four and six stratified by centre, KL score and index joint. The physician, the participant and all other staff involved in the trial will be masked to the participant’s treatment allocation. Concealed allocation is guaranteed by randomisation software that will assign subjects to treatment groups, matching placebo to colchicine and strict procedures for blinded and unblinded parties in the drug supply chain. The investigator will unblind the treatment allocation of a subject during the clinical trial only if unblinding is relevant to a subject’s safety. In case of unblinding, the investigator can open a physical envelope labelled with the participant’s treatment number. This envelope will contain information about the trial medication that was received. Other participants will not be unblinded. To assess the success of blinding, participants will be asked annually to indicate which group they believe they are in.

### Intervention

Colchicine Tiofarma 0.5 mg tablets will be orally administered with water once a day. If a dose is missed, it should be taken later during the day or skipped if noticed after 12 hours. A matching placebo is the comparator. Trial medication will be (temporarily) discontinued if health issues overrule treatment continuation as determined by the clinical site investigator or if one of the following drugs is prescribed for a given period: macrolide antibiotics (clarithromycin, erythromycin and azithromycin), antimycotics (ketoconazole, itraconazole and voriconazole), protease inhibitors and antiretroviral drugs (ritonavir, lopinavir, tipranavir, atazanavir, darunavir, indinavir, saquinavir and cobicistat), antiarrhythmic drugs (verapamil and diltiazem) and immunosuppressants (cyclosporine).

### Study procedures

Patients will be asked to complete online questionnaires using Castor EDC at enrolment, at baseline and every 3 months after that. Face-to-face contact will take place at enrolment, at baseline and every year during follow-up. The number of visits depends on the moment of inclusion and ranges between a minimum of five and a maximum of seven. Participants who discontinue the trial medication or withdraw from the study for any reason will continue to be followed for the primary outcome (knee or hip replacement). The study procedures are shown in online supplemental additional file 3.

#### Demographics

Data regarding age, sex, ethnicity, education level, profession, employment, smoking, alcohol, index joint, number and type of affected joints and duration of complaints will be collected at enrolment.

#### Anthropometrics

Height, weight and waist circumference will be assessed at baseline. Subsequent measures of weight and waist circumference will be taken during clinical visits. Body mass index will be calculated using weight and height.

#### Comorbidities

The following comorbid conditions will be documented at each clinical visit: heart disease (for example angina, heart attack or heart failure); high blood pressure; problems caused by a stroke; leg pain when walking due to poor circulation; lung disease (for example asthma, chronic bronchitis or emphysema); diabetes mellitus; kidney disease; diseases of the nervous system (for example Parkinson’s disease or multiple sclerosis); liver disease; cancer (within the last 5 years); depression; arthritis in the back or other condition affecting the spine; rheumatoid arthritis or another kind of arthritis in addition to OA.^28^

#### Joint replacement

Participants will be asked every 3 months about any knee or hip replacement surgery through a multiple-choice question. In case of an event, source documents will be collected for central adjudication.

#### OA diagnosis

Participants will be asked every 12 months about any new OA diagnosis in a joint other than the knee or hip through a yes/no question. In case of an event, source documents will be collected for central adjudication.

#### Cardiovascular events

Participants will be asked every 12 months about any cardiovascular event defined as myocardial infarction, peripheral artery disease, ischaemia-driven coronary revascularisation, ischaemic stroke or cardiovascular death through a multiple-choice question. In case of an event, source documents will be collected for central adjudication.

#### Pain medication

Participants will be asked every 3 months about any concomitant pain medication used, including prescription and over-the-counter medications.

#### Numeric Rating Scale

The Numeric Rating Scale (NRS) will assess pain levels during rest and during movement at baseline and every 3 months after that. The NRS consists of 11 numbers from 0 to 10, where 0 means no pain and 10 represents the most imaginable pain.

#### Western Ontario and McMaster Universities Arthritis Index

The Western Ontario and McMaster Universities Arthritis Index is designed to assess pain, stiffness and physical functioning in patients with knee or hip OA using 24 questions and will be assessed at baseline and every 6 months after that.^29^ Higher scores represent worse outcomes.

#### European Quality of Life 5-Dimensions 5-Level

The European Quality of Life 5-Dimensions 5-Level (EQ-5D-5L) measures quality of life at baseline and every 6 months after that using five levels of severity in terms of mobility, self-care, usual activities, pain/discomfort and anxiety/depression.^30^

#### iMTA Productivity Cost Questionnaire

The iMTA Productivity Cost Questionnaire (iPCQ) will assess loss of productivity due to OA or joint replacement surgery based on patient-reported absences from paid or unpaid labour at baseline and every 6 months after that.^31^

#### iMTA Medical Consumption Questionnaire

The iMTA Medical Consumption Questionnaire (iMCQ) will assess all relevant healthcare-related costs like outpatient visits to medical specialists, hospitalisations and paramedic care at baseline and every 6 months after that.^32^

#### Medication Adherence Report Scale-5

The Medication Adherence Report Scale-5 questionnaire will be assessed every 6 months to evaluate adherence to trial medication and consists of five self-reported adherence items about forgetting, changing dosage, stopping, skipping and taking less medication. Scores range from 5 to 25, with higher scores indicating better medication adherence.^33^

#### Pill count

Adherence to trial medication will be evaluated through pill counts once a year.

#### Radiography

Radiographic examinations include standard clinical radiographs of the index knee or hip at baseline if not made in the past 6 months and in a subgroup of 200 patients who have taken at least 80% of the study medication for a minimum of 2 years at the end of the study. Joint space narrowing will be assessed using automated software.

#### Blood samples

Blood samples will be drawn at enrolment, at baseline, after 1 year and at the end of the study to assess inflammation (hs-CRP) and safety (kidney function, liver function, creatine kinase (CK), erythrocytes, leucocytes, thrombocytes and, if anaemia is present, vitamin B12).

#### Adverse events

Abnormal CK, eGFR and ALAT values will be registered throughout the study period. The following AEs will be systematically assessed once a year: gastrointestinal, infectious, musculoskeletal and connective tissue disorders, cardiac disorders and neurological disorders.

### Study endpoints

The primary endpoint of this study is time from randomisation until the date of first documented knee or hip replacement, date of death, date of loss to follow-up or study end-date, whichever comes first, assessed up to 4.5 years.

Secondary endpoints include: (1) course of pain, (2) course of physical function, (3) course of joint space narrowing, (4) course of low-grade inflammation, (5) course of quality of life, (6) number of participants with and time to clinical or radiological onset of OA in a new joint group other than present at baseline, (7) number of participants using pain medication during the study, (8) onset of new cardiovascular events, including myocardial infarction, ischaemia-driven coronary revascularisation, ischaemic stroke, peripheral artery disease or cardiovascular death and (9) direct and indirect costs related to treatment and disease burden due to OA from randomisation until the date of first documented knee or hip replacement, date of death, date of loss to follow-up or study end-date, whichever comes first, assessed up to 4.5 years.

Harm-related endpoints include: (1) the number of (S)AEs, (2) the number of withdrawals due to (S)AEs and (3) changes in laboratory data (ie, serum creatinine, eGFR and ALAT) throughout the study.

### Sample size calculation

This trial is designed to accrue a minimum of 380 primary end-point events for the Cox proportional hazard model to achieve 80% power with an alpha of 0.05 and detect a HR of 0.75 (based on the exploratory results from the LoDoCo2 trial).^25^ To achieve this number of events, the number of required patients varies based on the baseline event rate and the inclusion rate (as with more inclusions at the beginning of the inclusion period, patients will on average have longer follow-up within the planned total study duration of 4.5 years). Therefore, an adaptive design that allows for a blinded re-estimation of the sample size based on the observed number of events and speed of inclusion using the R package BSSRed (Blinded Sample Size Reestimation For Event Driven Trials) will be applied.^34^ The required number of patients and duration of the inclusion period for various scenarios regarding baseline event rate and inclusion rate is shown in table 1. For all scenarios, the maximum study duration until administrative censoring was set to 4.5 years, with a dropout of 10% at 2 years based on data from the LoDoCo2 trial.^26^ Scenarios concerning primary event rates were based on a previous study on the proportion of knee or hip replacements after 2 years in a sample of patients consulting an orthopaedic surgeon without indication for replacement (25% event rate) and the estimated proportion of patients consulting their general practitioner receiving hip or knee replacement in 2 years (23% event rate).^35^ In addition, we added a scenario with a conservative estimate (20% event rate). Based on these assumptions, 1410 patients will enter the run-in phase and 1200 patients will be randomised. Sample size re-estimation is planned at the end of the planned inclusion period at 18 months and does not require correction for multiplicity as allocation remains blinded and no between-arm comparisons are performed.

**Table 1:** Estimation of the sample size and length of the inclusion period for varying inclusion rates (1000, 1200 and 1500) per 18 months (the planned inclusion period) and primary event rates (20%, 23% and 25%) per 2 years.

Inclusion rate per18 months	Primary event rate per 2 years		
	20%	23%	25%
1000	1504 in 27 months	1280 in 23 months	1168 in 21 months
1200	1398 in 20 months	1200 in 18 months	1134 in 17 months
1500	1334 in 16 months	1166 in 14 months	1084 in 13 months

### Statistical analysis

Descriptive statistics will be used to describe the study sample. For normally distributed continuous variables, means and SDs will be calculated. For non-normally distributed continuous variables, medians and IQRs will be reported. Categorical or dichotomous variables will be summarised using absolute numbers and percentages.

The primary analysis will be performed according to the intention-to-treat principle. Kaplan-Meier curves will be used to depict the time to first knee or hip replacement in the two treatment groups. HRs of treatment with colchicine versus placebo, with corresponding 95% CIs, will be obtained from Cox proportional hazards regression models with stratification by centre, KL score and index joint. Sensitivity analyses will be performed by adding covariates for change in body weight over time and cumulative use of pain medication. All randomised patients will be included in the analyses. In addition, a per-protocol analysis of the primary endpoint will be performed in patients who have taken at least 80% of the study medication for a minimum of 2 years.

For the secondary analyses, linear mixed models or generalised estimating equations with repeated measures will be used to estimate mean between-group differences and their 95% CIs for continuous and binary secondary outcomes. Participants will be included as random effect and treatment allocation as fixed effect factors. Missing data will be handled by the mixed model.

A cost-effectiveness analysis will be carried out alongside the trial comparing colchicine to placebo from a societal perspective. First, the EQ-5D-5L will assess the impact of both strategies on the quality of life, and the utility will be used to derive a quality-adjusted life year (QALY) estimate for each patient according to the trapezium rule. Second, volumes of OA-related care will be measured at the patient level using the iMCQ, and loss of productivity due to OA or joint replacement surgery will be estimated based on patient-reported absences from paid (or unpaid) labour measured with the iPCQ. To determine the cost prices for each volume of consumption, the standard cost prices from the ‘Dutch Guidelines for Cost Analyses’ and www.medicijnkosten.nl will be used. For units of care where no standard prices are available, real cost prices will be determined based on full cost pricing. Productivity losses will be valued through the friction cost method. Finally, the incremental cost-effectiveness ratio (ICER) will be calculated by dividing the difference in costs (medical and societal) by the difference in QALYs between the groups. The ICER, indicating the additional cost required to gain one QALY, can be compared with the willingness to pay value. Uncertainty in the ICER will be non-parametrically determined using bootstrap techniques (1000 replications).

No formal statistical testing will be conducted for the harm-related endpoints. AEs and SAEs will be presented as numbers and percentages per intervention arm. The relationship between AEs and SAEs with the trial treatment will be evaluated, and numbers and percentages of treatment-related AEs and SAEs will be presented per treatment arm. Deaths, AEs and SAEs resulting in treatment discontinuation will be reported.

### Harm-related considerations

Except for planned hospitalisations because of knee or hip replacements, the investigator will record all AEs and SAEs including the date of occurrence, a description of the event and its severity, duration and the actions taken. All AEs and SAEs will be followed until they have abated or a stable situation has been reached. Due to the size and duration of the trial, an independent Data and Safety Monitoring Committee consisting of one rheumatologist, one clinical epidemiologist and one pharmacist from the Sint Maartenskliniek will blindly review the trial’s progress including updated figures on recruitment and safety data biannually and will advise on optimal execution. No interim analysis will be done.

### Data management

Data will be collected and processed following the General Data Protection Regulation (EU) 2016/679. Data will be entered in Castor EDC, recorded on electronic case report forms and stored on a department server with automatic back-ups. Paper-based data will be stored in locked cabinets at each site. Data will be kept for 25 years.

Subjects will be identified by a study-specific subject number and/or code in the database. Names and other identifying details will not be included in any study data electronic file. The key to the code is safeguarded by principal investigators.

Data quality will be promoted by data checks. If missing, questionable or out-of-range values are identified, these will be queried and corrected if possible. If this is not possible, questionable or out-of-range values will be excluded from analyses.

Monitoring and quality assurance will be established according to the advice of the Dutch Federation of University Medical Centres (NFU). A qualified monitor of the Department of Research of the Sint Maartenskliniek will visit all participating centres before trial commencement and annually thereafter to check trial procedures, including data recording, verification of source data and safety assessments.

### Patient and public involvement

From the preapplication of the grant for this project, two patient representatives of the STAP (key to active participation) panel (a hospital-based patient panel of around 50 patient research partners with rheumatic diseases to support orthopaedic and rheumatology research (both clinical and preclinical) at Radboudumc and Sint Maartenskliniek) actively engaged in this project. During this phase, the relevance and feasibility of this project were assessed. Suggestions were provided about participant retention, medication adherence and the assessment of adverse effects. This resulted in insights about the order of questionnaires and the amount and type of contact with researchers. Information about adverse effects and interaction with other drugs has been added to the patient information form (PIF). Their request to investigate differences between men and women has been incorporated in the statistical analysis.

After grant approval, two additional patient representatives agreed to actively participate in this project: one member of the national patient organisation P-AL and one member of the STAP panel. They provided feedback on the PIF and evaluated the feasibility for patients by testing study procedures. During later research phases, patient representatives will be involved in drafting a questionnaire to assess elementary eligibility criteria if patients express their interest in participating following disseminated study information from national publicity related to our previously published finding of the LoDoCo2 trial, ReumaNederland or P-AL and promoting participant retention. In addition, patient representatives will give input about the wording of results from a patient perspective, write lay summaries and can coauthor scientific publications.

### Ethics and dissemination

The proposed trial has been registered with the EU Clinical Trials Register (2024-511359-16-00) and on clinicaltrials.gov (NCT06578182). The protocol has been approved by the Medical Ethics Review Committee East-Netherlands. Written informed consent will be obtained from all participants by the treating or research physician (online supplemental additional 4). A separate question in the informed consent form will ask for permission to store and reuse personal data and samples for further research. Findings will be presented at scientific meetings and published with the full trial protocol in a peer-reviewed scientific journal. Pseudonymised data will be made available on reasonable request.

## Supplementary material

10.1136/bmjopen-2024-098096 online supplemental file 1

10.1136/bmjopen-2024-098096 online supplemental file 2

10.1136/bmjopen-2024-098096 online supplemental file 3

10.1136/bmjopen-2024-098096 online supplemental file 4

## Data Availability

Data sharing not applicable as no datasets generated and/or analysed for this study.
