# Supplementary material for: Design of a randomised, placebo-controlled, double-blind multicentre study assessing the effect of colchicine on the incidence of knee or hip replacements in symptomatic knee or hip osteoarthritis: the ECHO trial
Source: BMJ Open. 2025 Apr 14;15(4):e098096. doi: 10.1136/bmjopen-2024-098096 (PMC11997832; doi:10.1136/bmjopen-2024-098096)
Supplement: online supplemental file 2 [file bmjopen-15-4-s002.docx]

|  | University hospital | Peripheral hospital | Tertiary hospital |
| --- | --- | --- | --- |
| Participating centres |  | Canisius Wilhelmina Hospital  Gelderse Vallei*  Noordwest Hospital Group Alkmaar*  Rijnstate*  Reinier Haga Medical Diagnostic Centre* | Sint Maartenskliniek  Reade* |
| Referral centres | Radboudumc | Bernhoven |  |

*expressed their willingness to participate but are not yet officially registered as centre
