# Supplementary material for: Design of a randomised, placebo-controlled, double-blind multicentre study assessing the effect of colchicine on the incidence of knee or hip replacements in symptomatic knee or hip osteoarthritis: the ECHO trial
Source: BMJ Open. 2025 Apr 14;15(4):e098096. doi: 10.1136/bmjopen-2024-098096 (PMC11997832; doi:10.1136/bmjopen-2024-098096)
Supplement: online supplemental file 3 [file bmjopen-15-4-s003.docx]

|  | **Enrolment** | **Baseline** | **Follow-up** | | | | | | | | | | | | | **Close-out** |
| --- | --- | --- | --- | --- | --- | --- | --- | --- | --- | --- | --- | --- | --- | --- | --- | --- |
| Timepoint in months | -1 | 0 | 3 | 6 | 9 | 12 | 15 | 18 | 21 | 24 | 27 | 30 | 33 | … | 51 | 36-54 |
| Eligibility screening | x |  |  |  |  |  |  |  |  |  |  |  |  |  |  |  |
| Informed consent | x |  |  |  |  |  |  |  |  |  |  |  |  |  |  |  |
| Open label colchicine |  |  |  |  |  |  |  |  |  |  |  |  |  |  |  |  |
| Allocation |  | x |  |  |  |  |  |  |  |  |  |  |  |  |  |  |
| Colchicine or placebo |  |  |  |  |  |  |  |  |  |  |  |  |  |  |  |  |
| Sociodemographics |  |  |  |  |  |  |  |  |  |  |  |  |  |  |  |  |
| Age | x |  |  |  |  |  |  |  |  |  |  |  |  |  |  |  |
| Sex | x |  |  |  |  |  |  |  |  |  |  |  |  |  |  |  |
| Ethnicity | x |  |  |  |  |  |  |  |  |  |  |  |  |  |  |  |
| Education level | x |  |  |  |  |  |  |  |  |  |  |  |  |  |  |  |
| Employment | x |  |  |  |  |  |  |  |  |  |  |  |  |  |  |  |
| Profession | x |  |  |  |  |  |  |  |  |  |  |  |  |  |  |  |
| Smoking | x |  |  |  |  |  |  |  |  |  |  |  |  |  |  |  |
| Alcohol | x |  |  |  |  |  |  |  |  |  |  |  |  |  |  |  |
| Height | x |  |  |  |  |  |  |  |  |  |  |  |  |  |  |  |
| Weight | x |  |  |  |  | x |  |  |  | x |  |  |  | x |  | x |
| Waist circumference | x |  |  |  |  | x |  |  |  | x |  |  |  | x |  | x |
| Disease characteristics |  |  |  |  |  |  |  |  |  |  |  |  |  |  |  |  |
| Index joint | x |  |  |  |  |  |  |  |  |  |  |  |  |  |  |  |
| Affected joints | x |  |  |  |  |  |  |  |  |  |  |  |  |  |  |  |
| Duration of complaints | x |  |  |  |  |  |  |  |  |  |  |  |  |  |  |  |
| Joint replacement |  |  | x | x | x | x | x | x | x | x | x | x | x | x | x | x |
| New OA diagnosis |  |  |  |  |  | x |  |  |  | x |  |  |  | x |  | x |
| Comorbidities | x | x |  |  |  | x |  |  |  | x |  |  |  | x |  | x |
| Cardiovascular events |  | x |  |  |  | x |  |  |  | x |  |  |  | x |  | x |
| Questionnaires |  |  |  |  |  |  |  |  |  |  |  |  |  |  |  |  |
| Pain medication |  |  | x |  | x |  | x |  | x |  | x |  | x |  | x |  |
| MARS-5 |  | x |  | x |  | x |  | x |  | x |  | x |  | x |  | x |
| NRS pain | x | x | x | x | x | x | x | x | x | x | x | x | x | x | x | x |
| WOMAC |  | x |  | x |  | x |  | x |  | x |  | x |  | x |  | x |
| EQ-5D-5L |  | x |  | x |  | x |  | x |  | x |  | x |  | x |  | x |
| iPCQ |  | x |  | x |  | x |  | x |  | x |  | x |  | x |  | x |
| iMCQ |  | x |  | x |  | x |  | x |  | x |  | x |  | x |  | x |
| Pill count |  | x |  |  |  | x |  |  |  | x |  |  |  | x |  | x |
| X-ray* |  | x |  |  |  |  |  |  |  |  |  |  |  |  |  | x |
| Blood sampling | x | x |  |  |  | x |  |  |  |  |  |  |  |  |  | x |

Hospital visits are indicated in blue and tele contact moments are indicated in yellow. Since the study end date is approximately the same for all patients and the inclusion period is planned to last 1.5 years, the trial duration for an individual patient ranges between 3 and 4.5 years depending on the time of inclusion. *If not made in the past 6 months
