# Supplementary material for: Design of a randomised, placebo-controlled, double-blind multicentre study assessing the effect of colchicine on the incidence of knee or hip replacements in symptomatic knee or hip osteoarthritis: the ECHO trial
Source: BMJ Open. 2025 Apr 14;15(4):e098096. doi: 10.1136/bmjopen-2024-098096 (PMC11997832; doi:10.1136/bmjopen-2024-098096)
Supplement: online supplemental file 4 [file bmjopen-15-4-s004.pdf]

Appendix E: Informed consent form – subject

Belonging to The effect of colchicine on the number of knee- and hip prostheses (ECHO)

- I have read the information sheet. I was able to ask questions. My questions have been answered well enough. I had enough time to decide if I wanted to take part.
- I know that taking part is voluntary. I also know that at any time I can decide not to take part in the study. Or to stop taking part. I do not have to explain why.
- I give the investigator consent to inform my doctor and/or specialist who treats me that I am taking part in this study.
- I give consent to request information from my doctor and/or specialist treating me about osteoarthritis.
- I give consent to give my doctor or specialist information about accidental discoveries made during the study that are important for my health.
- I give consent to collect and use my data and/or body material. The investigators only do this to answer the question of this study. And to register the medicinal product.
- I know that some people will be able to see all of my data to review the study. These people are mentioned in this information sheet. I give consent to let them see my data for this review.
- I know that I cannot get pregnant/cannot get my partner pregnant during the study and until 6 months after stopping the trial medication.
- The investigator discussed with me how I can best prevent becoming pregnant/my partner from becoming pregnant.
- Please tick yes or no in the table below.

I give consent to store my data to use for other research, as stated in the information sheet.	Yes ☐	No☐
I give consent to have my (remaining) body material stored for use in other research, as stated in the information sheet. The body material is stored for this purpose for another 10 years.	Yes ☐	No☐
I give consent to ask me after this study if I want to participate in a follow-up study.	Yes ☐	No☐
I give consent to let me know after the study which treatment I received/in which group I was.	Yes ☐	No☐

- I want to take part in this study.

My name is (subject): ………………………………..
Signature: ………………………   Date : __/__/__

I declare that I have fully informed this subject about the study mentioned.

If any information becomes known during the study that could influence the subject's consent, I will let this subject know in good time.

Investigator name (or their representative): ....
Signature:……………………… Date: __/__/__

The study subject will receive a complete information sheet, together with a signed version of the consent form.
