# Supplementary material for: Design of a randomised, placebo-controlled, double-blind multicentre study assessing the effect of colchicine on the incidence of knee or hip replacements in symptomatic knee or hip osteoarthritis: the ECHO trial
Source: BMJ Open. 2025 Apr 14;15(4):e098096. doi: 10.1136/bmjopen-2024-098096 (PMC11997832; doi:10.1136/bmjopen-2024-098096)
Supplement: Supplementary file 5 [file bmjopen-2024-098096.reviewer_comments.pdf]

PEER REVIEW HISTORY

BMJ Open publishes all reviews undertaken for accepted manuscripts. Reviewers are asked to complete a checklist review form and are provided with free text boxes to elaborate on their assessment. These free text comments are reproduced below.

ARTICLE DETAILS

Title (Provisional)

Design of a randomised, placebo-controlled, double-blind multicentre study assessing the Effect of ColcHicine on the incidence of knee or hip replacements in symptomatic knee or hip Osteoarthritis: the ECHO trial

Authors

Heijman, Michelle W.J.; van den Ende, Cornelia HM; Cornel, Jan; Smolders, José M.H.; Schers, Henk; Kievit, Wietske; Koeter, Sander; van den Bemt, Bart; Popa, Calin D.

VERSION 1 - REVIEW

**Reviewer**	**1**
**Name**	**Pascart , Tristan**
**Affiliation**	**Lille Catholic Hospitals**
**Date**	**30-Dec-2024**
**COI**	**None**

I read with interest the protocol of the ambitious ECHO double-blind trial of colchicine in knee OA. I have some minor comments and congratulate the investigators for this project.

Introduction

I think making some refernce to the fact that colchicine has been used above all (and proved to be effective) in acute crystal-induced inflammation (AGREE and CONTACT trials for gout DOI: 10.1002/art.27327 and doi: 10.1136/annrheumdis-2019-216154, and COLCHICORT for CPPD disease doi: 10.1016/S2665-9913(23)00165-0), and for gout flare prophylaxis (doi: 10.1136/ard-2023-224154 and DOI: 10.1136/ard-2023-224731). This is particularly important as the potential effect of colchicine in OA may be mediated by a role of calcium crystals in joint inflammation and cartilage degradation (doi: 10.1016/S2665-9913(24)00122-X and https://doi.org/10.3390/gucdd2020010)

Methods

Why was eGFR limited at >50 in the inclusion criteria and not down to 30 ? particularly as the 1st run-in month of the trial would secure all participants as tolerating well colchicine ?

Why didn’t the author include a stratification on chondrocalcinosis on imaging ?

Did the authors plan some biobanking (including DNA and RNA seq)?

**Reviewer**	**2**
**Name**	**Samuels, Jonathan**
**Affiliation**	**NYU Langone Health, rheum**
**Date**	**14-Jan-2025**
**COI**	**None**

The authors present a description of a prospective randomized double-blind placebo-controlled study to o consider the utility of colchicine as a treatment for knee/hip osteoarthritis, with the primary outcome being rates of total joint arthroplasty. Assessment of this manuscript entails evaluation of the methods and planned statistical analysis, as there are not any results or discussion yet. The authors do a nice job framing the question by describing prior trials in colchicine and OA - as well as post-hoc consideration of colchicine used for other diseases (LoDoCo2), and the CANTOS trial.

The methods should answer a few questions:

1) If patients are only entered if they tolerate the run-in phase of open label colchicine, won't they know if they are subsequently getting placebo? Is there a better method to prevent this bias and potential dropout from the placebo arm ?

2) Please justify the 3-4.5 year duration of medication and assessment. How did you arrive at this ?

3) Why don't you have dosing arms at 0.5 QD vs BID vs placebo, instead of just QD ? Perhaps you could overcome this with BID dosing in hte run-in phase ?

4) If TKR/THR is the primary outcome, how do you justify overlooking the many other reasons why someone may not undergo this surgery - beyond the degree of OA progression such as cobmorbidiies> poor surgical candidate, fear of surgery /etc. ? Maybe you assume it will be similar for both groups, but do you correct for this ?

5) statisticans will need to review the sample size calculations and statistical analyses proposed here.

**Reviewer**	**3**
**Name**	**Dack, Kyle**
**Affiliation**	**University of Bristol, MRC Integrative Epidemiology Unit**
**Date**	**13-Feb-2025**

COI **None**

This protocol includes a thorough and transparent analysis plan. It seems a little vague concerning the sample size and dates, but these are minor points which can be resolved with a few extra lines of information.

Introduction

1. Line 64-65. It is worth clarifying that this is the global increase.

2. Line 89-93. This sentence is long and hard to follow, suggest splitting. This will also enable the referencing to be more specific. The paragraph heading stated that results from these four trials were inconsistent – but how? It would useful to very briefly report the results, e.g. two reported a significant reduction, whereas two did not.

Methods

3. Line 130. 1dd is not defined, but since it is only used once I suggest replacing with the full phrase “once per day”.

4. Line 134. The trial duration is described in figure 1 and the participant journey in table 1, but the key dates do not seem to me stated in the text itself. It would improve the clarity of the paper if some of the main study dates were described – including the months, because 2024 to 2029 is a little vague.

5. Line 292. “to first incident knee or hip replacement” seems redundant because it is repeated on the next line.

6. Sample size: several scenarios are estimated, and the authors state that sample size re-estimation will be performed. However, I do not see it explicitly stated what is the preliminary total sample size. From figure 1 it appears to be 1410?

7. Line 345-346. Will trial centre be adjusted for? Centre is stratified in the main analysis which suggests that geographical heterogeneity may be expected – is this also explored/adjusted for in secondary analyses?

8. Figure 1. Suggest replacing the years with the full dates to improve precision. The analysis has 600 per arm while followup has 538 – I assume due to ITT, but I suggest clarifying this, e.g. replace “analysis” with “ITT analysis” if this is the reason.

VERSION 1 - AUTHOR RESPONSE

Reviewer 1 (Prof. Tristan Pascart)

1. **I read with interest the protocol of the ambitious ECHO double-blind trial of colchicine in knee OA. I have some minor comments and congratulate the investigators for this project.**
   We appreciate your congratulations and the time taken to review our manuscript.

Introduction

2. **I think making some reference to the fact that colchicine has been used above all (and proved to be effective) in acute crystal-induced inflammation (AGREE and CONTACT trials for gout DOI: 10.1002/art.27327 and doi: 10.1136/annrheumdis-2019-216154, and COLCHICORT for CPPD disease doi: 10.1016/S2665-9913(23)00165-0), and for gout flare prophylaxis (doi: 10.1136/ard-2023-224154 and DOI: 10.1136/ard-2023-224731). This is particularly important as the potential effect of colchicine in OA may be mediated by a role of calcium crystals in joint inflammation and cartilage degradation (doi: 10.1016/S2665-9913(24)00122-X and https://doi.org/10.3390/gucdd2020010)**
   We thank the reviewer for the suggestion and have rephrased lines 89-91.
   Methods
3. **Why was eGFR limited at >50 in the inclusion criteria and not down to 30? Particularly as the 1st run-in month of the trial would secure all participants as tolerating well colchicine?**
   The limit for eGFR was set at >50 from the safety perspective, as this is the standard cut-off value used in the leaflet of colchicine on the Dutch market and the pharmacovigilance software used in the Netherlands by prescribers and pharmacists.
4. **Why didn't the author include a stratification on chondrocalcinosis on imaging?**
   To prevent over-stratification (Kernan et al., 1999).
5. **Did the authors plan some biobanking (including DNA and RNA seq)?**
   Serum samples will be stored for future use, but measurements are not yet specified. Unfortunately, we did not have sufficient financial resources to collect blood using PAXgene tubes to perform genetic studies.

<u>**Reviewer 2 (Dr. Jonathan Samuels)**</u>

The authors present a description of a prospective randomized double-blind placebo-controlled study to o consider the utility of colchicine as a treatment for knee/hip osteoarthritis, with the primary outcome being rates of total joint arthroplasty. Assessment of this manuscript entails evaluation of the methods and planned statistical analysis, as there are not any results or discussion yet. The authors do a nice job framing the question by describing prior trials in colchicine and OA - as well as post-hoc consideration of colchicine used for other diseases (LoDoCo2), and the CANTOS trial.

Thank you for your considerate assessment of our study and the positive comment.

The methods should answer a few questions:

1. **If patients are only entered if they tolerate the run-in phase of open label colchicine, won't they know if they are subsequently getting placebo? Is there a better method to prevent this bias and potential dropout from the placebo arm?**
   We understand the reviewer's concern. However, the effect of colchicine after 30 days is negligible (Das et al., 2003). Furthermore, based on data from the LoDoCo2 trial (Nidorf et al., 2020), we do not expect unblinding to occur due to the recognizability of side effects. In this trial, 6,528 patients received colchicine during a 30-day run-in phase. A total of 1,006 patients were not randomized, with side effects being the reason in 611 cases. Among the 5,522 patients who were randomized, 95 in the colchicine group and 93 in the placebo group discontinued medication early due to side effects. Other large studies on colchicine,

such as the COLCOT (Tardif et al., 2019) and COPS (Tong et al., 2020) trials, also show no differences in the reported rates of side effects.

2. **Please justify the 3-4.5 year duration of medication and assessment. How did you arrive at this?**
   The minimum follow-up duration was conservatively estimated at 3 years, based on the post-hoc analysis of the LoDoCo2 trial (Heijman et al., 2023). This analysis showed that the effect of colchicine treatment increases after 2 years. Given an inclusion period of 1.5 years, the maximum follow-up duration will be 4.5 years.
3. **Why don't you have dosing arms at 0.5 QD vs BID vs placebo, instead of just QD? Perhaps you could overcome this with BID dosing in the run-in phase?**
   Including 1:1:1 randomisation instead of 1:1 increases the overall sample size, making it unfeasible to recruit the required number of patients within the maximum requestable budget. The low-dose regimen of 0.5mg QD was chosen over 0.5mg BID as it minimizes the potential for side effects while remaining effective (Heijman et al., 2023).
4. **If TKR/THR is the primary outcome, how do you justify overlooking the many other reasons why someone may not undergo this surgery - beyond the degree of OA progression such as cobmorbidiies> poor surgical candidate, fear of surgery /etc. ? Maybe you assume it will be similar for both groups, but do you correct for this?**
   Patients with any absolute contraindication for knee or hip replacement, as assessed by a physician, will be excluded at baseline. Additionally, patients who refuse surgery based on personal convictions and beliefs will also be excluded.
5. **Statisticans will need to review the sample size calculations and statistical analyses proposed here.**
   At the time of the grant application and METC approval, a statistician and epidemiologist with a strong background in statistics assisted in developing the sample size calculation and statistical analysis.

<u>**Reviewer 3 (Dr. Kyle Dack)**</u>

1. **This protocol includes a thorough and transparent analysis plan. It seems a little vague concerning the sample size and dates, but these are minor points which can be resolved with a few extra lines of information.**
   We thank the reviewer for the time and effort taken to review our manuscript.
   Introduction
2. **Line 64-65. It is worth clarifying that this is the global increase.**
   We appreciate the suggestion and added this in line 70-71
3. **Line 89-93. This sentence is long and hard to follow, suggest splitting. This will also enable the referencing to be more specific. The paragraph heading stated that results from these four trials were inconsistent – but how? It would useful to very briefly report the results, e.g. two reported a significant reduction, whereas two did not.**
   We agree with the reviewer and have split the sentence in line 95-102. The sentence about inconsistent results was deleted as it was unclear that this sentence referred to lines 93-117.
   Methods
4. **Line 130. 1dd is not defined, but since it is only used once I suggest replacing with the full phrase “once per day”.**
   We replaced 1dd by once daily in line 138-139.

5. **Line 134. The trial duration is described in figure 1 and the participant journey in table 1, but the key dates do not seem to me stated in the text itself. It would improve the clarity of the paper if some of the main study dates were described – including the months, because 2024 to 2029 is a little vague.**
   Study dates, including the months, were added in lines 141-144.
6. **Line 292. "to first incident knee or hip replacement" seems redundant because it is repeated on the next line.**
   We thank the reviewer for the suggestion and deleted "to first incident knee or hip replacement" in line 301.
7. **Sample size: several scenarios are estimated, and the authors state that sample size re-estimation will be performed. However, I do not see it explicitly stated what is the preliminary total sample size. From figure 1 it appears to be 1410?**
   We apologize for being unclear. A total of 1410 patients will enter the run-in phase and 1200 patients will be randomized. This information is added in lines 332-333.
8. **Line 345-346. Will trial centre be adjusted for? Centre is stratified in the main analysis which suggests that geographical heterogeneity may be expected – is this also explored/adjusted for in secondary analyses?**
   No, we stratify by centre in the main analysis because the reasons for performing TKR/THR may vary between centres. However, this does not apply to the secondary variables.
9. **Figure 1. Suggest replacing the years with the full dates to improve precision. The analysis has 600 per arm while followup has 538 – I assume due to ITT, but I suggest clarifying this, e.g. replace "analysis" with "ITT analysis" if this is the reason.**
   Months were added to the years and intention to treat was added to analysis in Figure 1.

VERSION 2 - REVIEW

**Reviewer**	**2**
**Name**	**Samuels, Jonathan**
**Affiliation**	**NYU Langone Health, rheum**
**Date**	**19-Mar-2025**
**COI**	

Nice job answering the comments. While there aren't data yet, it is helpful to publish these methods.
